# A Member of the Nuclear Receptor Superfamily, Designated as NR2F2, Supports the Self-Renewal Capacity and Pluripotency of Human Bone Marrow-Derived Mesenchymal Stem Cells

**DOI:** 10.1155/2016/5687589

**Published:** 2015-12-13

**Authors:** Ni Zhu, Huafang Wang, Binsheng Wang, Jieping Wei, Wei Shan, Jingjing Feng, He Huang

**Affiliations:** Bone Marrow Transplantation Center, The First Affiliated Hospital, Zhejiang University School of Medicine, Hangzhou, Zhejiang 310003, China

## Abstract

Mesenchymal stem cells are characterized with self-renewal capacity and pluripotency. NR2F2 is a nuclear receptor that has been detected in the mesenchymal compartment of developing organs. However, whether NR2F2 plays a role in the stemness maintenance of mesenchymal stem cells has not been explored yet. In this study, we investigated the function of NR2F2 in bone marrow-derived mesenchymal stem cells via shRNA-mediated knock-down of NR2F2. The suppression of NR2F2 impaired the colony-forming efficacy of mesenchymal stem cells. The inhibition of colony-forming capacity may be attributed to the acceleration of senescence through upregulation of P21 and P16. The downregulation of NR2F2 also suppressed both osteogenic and adipogenic differentiation processes. In conclusion, NR2F2 plays an important role in the stemness maintenance of bone marrow-derived mesenchymal stem cells.

## 1. Introduction

Mesenchymal stem cells (MSCs) that can be isolated from various tissues such as bone marrow, adipose tissue [1], and umbilical cord blood [2] are a type of stem cells owning the capacity of self-renewal and multipotential differentiation. MSCs have been reported to own the abilities to form colony-forming unit-fibroblasts (CFU-F) and differentiate into osteoblasts, adipocytes, chondrocytes, and myoblasts [3]. In addition, MSCs exhibit immunomodulatory effects [4] and play effective roles in tissue repair. Thus, MSCs have prospects in cell-based therapies of various diseases, especially in the field of regenerative medicine. Serials of clinical trials have revealed inspiring results that MSCs infusion could improve cardiac [5] and hepatic functions [6] and benefit the outcome of graft-versus-host disease (GVHD) patients [7, 8]. The biological properties of MSCs are the fundamentals for clinical researches. Therefore it is of great importance to explore the biological characteristics of MSCs, especially the regulatory mechanisms of stemness.

NR2F2 (nuclear receptor subfamily 2, group F, member 2, or chicken ovalbumin upstream promoter-transcription factor II), a member of the nuclear receptor superfamily, which is widely expressed in the mesenchymal compartment of developing organs [9], has been demonstrated to have effects on the regulation of MSC differentiation [10] and mouse embryogenesis [11]. Through knock-down experiments in vitro and knock-out experiments in vivo, Xie et al. demonstrated that NR2F2 may induce adipogenic differentiation and suppress osteoblastic differentiation of MSCs via activation of PPAR*γ* and Sox9 expression as well as inhibition of Wnt signaling pathway and Runx2 [10].

To our knowledge, whether NR2F2 takes part in the self-renewal of MSCs has not been explored yet. Therefore, this study was undertaken to investigate the role of NR2F2 in the maintenance and support of BM-MSCs stemness.

## 2. Material and Methods

### 2.1. Generation of BM-MSCs

Human bone marrow samples from normal donors were collected for generation of mesenchymal stem cells. Bone marrow mononuclear cells were isolated by density gradient centrifugation and cultured in low-glucose Dulbecco's modified Eagle's medium (DMEM, 10-014-CVR, Corning) supplemented with 10% fetal bovine serum (FBS, 10099-141, Gibco) at 37°C and 5% CO_2_ in a humidified incubator. The medium was replaced after the first 48 hours and changed every 3 days later. The adherent cells were passaged when 90% confluence was reached. The BM-MSCs of passages 2–6 were used in the following assays.

### 2.2. Characteristics of BM-MSCs

BM-MSCs of passages 3–5 or transfected BM-MSCs were harvested and incubated with anti-CD90-FITC (11-0909), anti-CD90-PE (12-0909), anti-CD105-PE (12-1057), anti-CD73-APC (17-0739), anti-CD45-FITC (11-9459), anti-CD45-PE (12-9459), anti-CD34-PE (12-0349), anti-CD19-APC (17-0199), and anti-CD11b-PE (12-0113, eBioscience) antibodies at 4°C for 30 minutes. Results were detected by using an FC 500 MCL Flow Cytometer (Beckman Coulter). Appropriate isotype-matched antibodies (eBioscience) were applied as controls.

### 2.3. shRNA-Mediated Knock-Down of NR2F2

In order to acquire decreased expression of NR2F2 in BM-MSCs, the specified shRNA, which was reported previously [11] (5′-AGGTAACGTGATTGATTCAGTATCTTA-3′), was cloned into pGLV3 plasmid (GenePharma). A scrambled sequence (5′-TTCTCCGAACGTGTCACGT-3′) was also cloned into pGLV3 as the negative control. Lentiviral supernatant was produced by cotransfecting 293T cells with pMD2.G plasmid, psPAX2 plasmid, and shRNA-plasmid with the calcium phosphate transfection kit (BW11002, Biowit Technologies) according to manufacturer's instructions. Viral suspension was collected, filtered, and added to BM-MSCs directly after removal of the medium. Cells were incubated with viral suspension and virus was replaced with fresh medium after 10 hours. The GFP expression was assessed by using an inversed fluorescent microscope. The positive expression of GFP was employed to evaluate transfection efficiency.

### 2.4. Colony-Forming Unit-Fibroblast Assay

The transfected BM-MSCs (including the knock-down group and the negative control group) were harvested by digesting using 0.25% trypsin-EDTA solution (25200-056, Gibco). The cells were seeded in 6-well plates (CLS3516, Corning) with a density of 50 cells per cm^2^ in triplicate and incubated in DMEM with 10% FBS. After 14 days, cells were fixed in methanol and stained with 2% crystal violet for 10 min after removal of medium and washing with PBS. Colonies consisting of more than 50 cells were calculated after removal of redundant stain with PBS.

### 2.5. MTT (3-(4,5-Dimethyl-2-thiazolyl)-2,5-diphenyltetrazolium Bromide) Assay

Transfected BM-MSCs were seeded on a 96-well plate at a concentration of 2 × 10^3^ per well. Three parallel wells of cells were applied for each group. After incubation of 2 days, 4 days, or 6 days, 10 *μ*L MTT (LK-MTT001, Liankebio) was added. The medium was discarded after 4 hours. The OD values were evaluated by a microplate reader (SpectraMax M5, Molecular Devices) at 570 nm after adding 100 *μ*L DMSO.

### 2.6. Osteogenic and Adipogenic Differentiation

The transfected BM-MSCs at passages 3–5 were cultured in osteogenic differentiation medium (HUXMA-90021, Cyagen) for 21 days, with medium changed every 3 days. The formation of calcium nodules stained with Alizarin Red (HUXMA-90021, Cyagen) was employed to assess the osteogenic differentiation. For the induction of adipogenic differentiation, the transfected BM-MSCs at passages 3–5 were cultured in adipogenic differentiation medium A (HUXMA-90031, Cyagen) for the first 3 days. Later the cells were cultured in adipogenic differentiation medium B (HUXMA-90031, Cyagen). After 24 hours, medium B was replaced with adipogenic differentiation medium A. Cells were finally cultured in adipogenic differentiation medium B for additional 7 days with medium changed every 3 days after three cycles of maintenance. Cells were stained with Oil red O solution (HUXMA-90031, Cyagen) for the evaluation of adipogenic differentiation.

### 2.7. Senescence Analysis

Transfected BM-MSCs were seeded in 6-well plates. Medium was removed when cells achieved 50% confluence. Cells were rinsed with PBS and fixed with 1x fixative solution provided by senescence *β*-galactosidase staining kit (9860, Cell Signaling Technology) for 15 minutes. Fresh *β*-galactosidase staining solution was prepared according to manufacturer's instructions. Cells in each well were stained with 1 mL staining solution after being washed with PBS for 2 times. The process of staining was accomplished after incubation at 37°C in a dry incubator for 16 hours. The *β*-galactosidase positive cells were considered as senescent cells and counted in at least 4 randomly chosen fields.

### 2.8. Apoptosis Assay

Transfected BM-MSCs were harvested and washed with cold PBS for 2 times. Cells were resuspended in 100 *μ*L 1x Binding Buffer provided in apoptosis detection kit (559763, BD Pharmingen). Cells were incubated with Annexin V-PE antibody and 7-AAD (559763, BD Pharmingen) at room temperature for 15 minutes. Results were detected by using a FC 500 MCL Flow Cytometer (Beckman Coulter). Cells that were Annexin-V-positive and 7-AAD-negative were considered to be early apoptotic. Cells were considered to be late apoptotic when both Annexin-V and 7-AAD were positive.

### 2.9. Quantitative Reverse Transcription PCR (qRT-PCR)

Reverse transcription was carried out with a PrimeScript RT reagent kit with gDNA Eraser kit (RR047A, Takara) after total cellular RNA of transfected BM-MSCs extracted by using Trizol reagent (15596-026, Life Technologies). Quantitative PCR was performed by using SYBR Premix Ex Taq II (RR420A, Takara) according to manufacturer's instructions with a LightCycler system (Roche Diagnostics). Samples were all prepared in triplicate. The expression of glyceraldehydes 3-phosphate dehydrogenase (*GAPDH*) was detected for normalization. The primers used were as follows:* NANOG* forward 5′-TGC­CTC­ACA­CGG­AGA­CTG­T-3′, reverse 5′-CTT­TGG­GAC­TGG­TGG­AAG­AAT-3′;* OCT4* forward 5′-GTA­TTC­AGC­CAA­ACG­ACC­ATCT-3′, reverse 5′-GCT­TCC­TCC­ACC­CAC­TTC­T-3′;* SOX2* forward 5′-GGA­GAG­AGA­AAG­AAA­GGG­AGA­GA-3′, reverse 5′-GCC­GCC­GAT­GAT­TGT­TAT­TAT-3′; alkaline phosphatase (*ALPL*) forward 5′-TGC­TCT­GCG­CAG­GAT­TGG­AAC­A-3′, reverse 5′-AGG­CAG­GTG­CCA­ATG­GCC­AGT­A; binding sialoprotein (*BSP*) forward 5′-GGA­GGA­GGA­AGA­AGA­GGA­GAC­T-3′, reverse 5′-CCC­AGT­GTT­GTA­GCA­GAA­AGT­G-3′; runt-related transcription factor 2 (*RUNX2*) forward 5′-GCA­GTT­CCC­AAG­CAT­TTC­AT-3′, reverse 5′-CTG­GCG­GGG­TGT­AAG­TAA­AG-3′; lipoprotein lipase (*LPL*) forward 5′-TTA­CCC­AGT­GTC­CGC­GGG­CT-3′, reverse 5′-AGA­CGA­CTC­GGG­GCT­TCT­GCA-3′; peroxisome proliferator-activated receptor-*γ* (*PPAR-γ*) forward 5′-ATC­AAG­AAG­ACG­GAG­ACA­GAC­A-3′, reverse 5′-AAC­TGG­AAG­AAG­GGAA­ATG­TTG-3′;* P21* forward 5′-AGG­GGA­CAG­CAG­AGG­AAG­AC-3′, reverse 5′-GGC­GTT­TGG­AGT­GGT­AGA­AA-3′;* P16* forward 5′-GTG­CCA­CAT­TCG­CTA­AGT­GCT-3′, reverse 5′-GAC­CCT­GTC­CCT­CAA­ATC­CTC­T-3′;* GAPDH* forward 5′-AGA­AGG­CTG­GGG­CTC­ATT­TG-3′, reverse 5′-AGG­GGC­CAT­CCA­CAG­TCT­TC-3′.

### 2.10. Western Blot Analysis

The transfected BM-MSCs were harvested by digesting using 0.25% trypsin-EDTA solution. Cells were lysed on ice for 1 hour in RIPA lysis buffer (AR0105, Boster) with PMSF solution (AR1179, Boster) after being washed once with cold PBS. Cell lysates were centrifuged at 12,000 ×g for 5 minutes at 4°C. The supernatant was incubated at 100°C for 10 minutes after appropriate amount of Loading Buffer was added. The prepared protein extracts were loaded onto a 10% polyacrylamide gel containing SDS, electrophoresed, and transferred to a nitrocellulose membrane. The membrane was blocked in 5% nonfat milk at room temperature for 1 hour, incubated with primary antibodies overnight, and then incubated with fluorescent secondary antibodies (926-65010 and 926-65020, LI-COR) at room temperature for 1 hour. The specific antibodies for NR2F2 (6434) and Caspase-3 (9665) were from Cell Signaling Technology. The specific antibody for P21 (ab109520) was from Abcam and the antibody for GAPDH (Mab5079) was from Multibioscience. Immunoreactive bands were visualized using an Odyssey infrared imaging system (LI-COR). GAPDH protein was used to ensure equivalent loading of protein samples.

### 2.11. Statistical Analysis

Statistical analysis was performed using SPSS 19.0 and differences between mean values were evaluated using the 2-tailed Student's *t*-test. A *P* value < 0.05 (*∗*) was defined as statistical significance.

## 3. Results

### 3.1. Characterization of BM-MSCs

**Figure 1:**
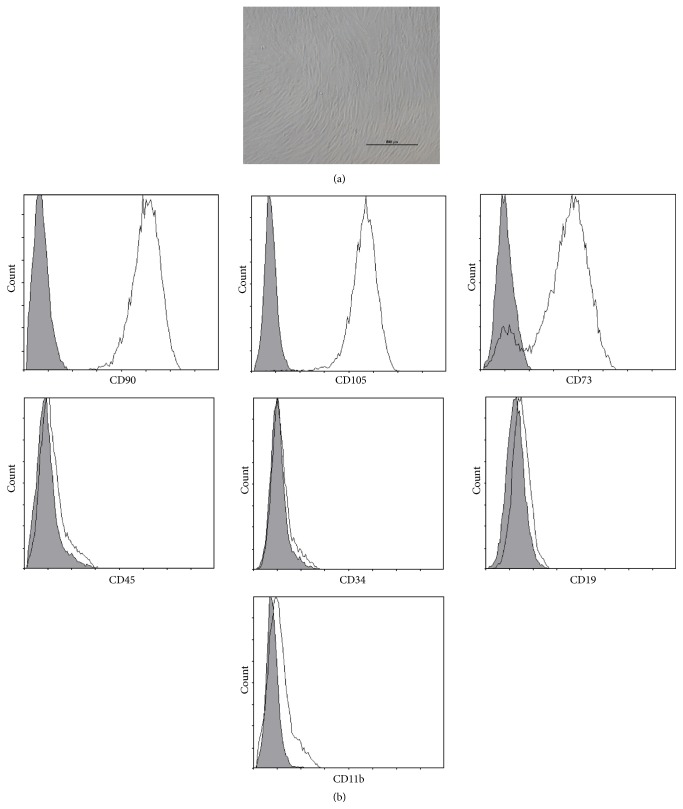
Characteristics of BM-MSCs. (a) Representative morphology of BM-MSCs. Scale bar = 500 *μ*m. (b) Representative flow cytometric characterization of cell surface markers expressed on BM-MSCs. Isotypic controls were represented by the gray filled histograms.

**Figure 2:**
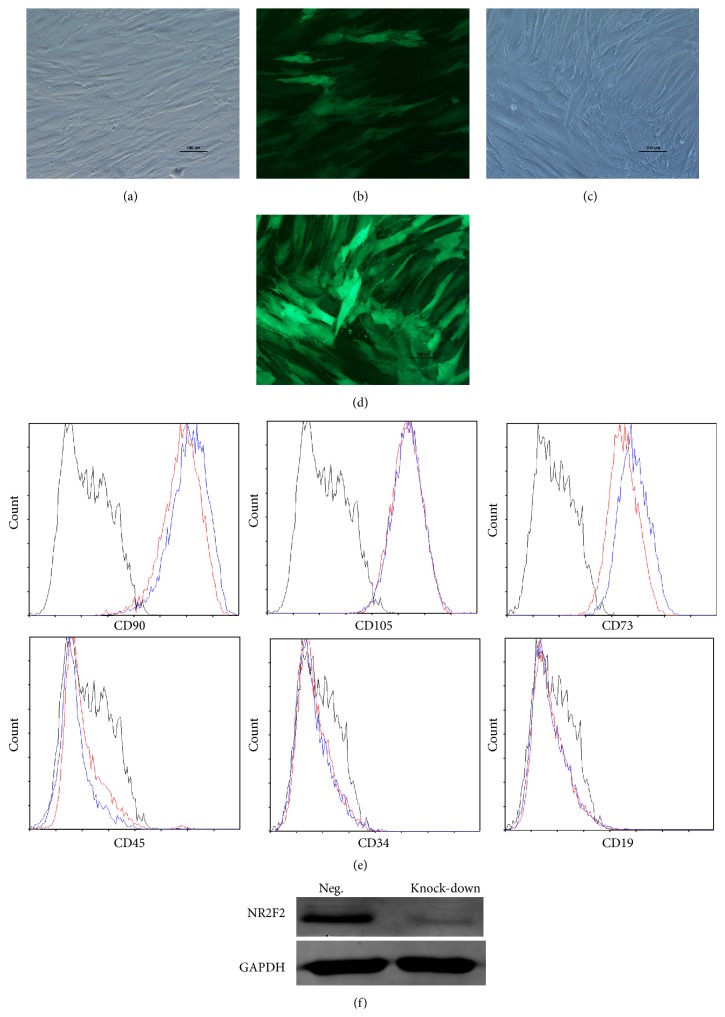
Characteristics of transfected BM-MSCs. (a) Representative morphology of transfected BM-MSCs in negative control group. (b) More than 90% of BM-MSCs expressed GFP in negative control group. (c) Representative morphology of transfected BM-MSCs in knock-down group. (d) More than 90% of BM-MSCs expressed GFP in knock-down group. (e) Representative flow cytometric characterization of cell surface markers expressed on transfected BM-MSCs. Isotypic controls were represented by black line. The red line represented the negative control group and the blue line represented the knock-down group. (f) The knock-down of NR2F2 was confirmed by western blot analysis.

BM-MSCs presented a spindle shape with fibroblast-like morphology (Figure 1(a)). As shown in Figure 1(b), BM-MSCs were positive for CD90 and CD105, mostly positive for CD73, but were negative for hematopoietic markers like CD34 and CD45. In addition, cells were negative for CD19, a marker of B-lineage origin and CD11b, which is expressed on myeloid cells and natural killer cells. We employed a specified shRNA to acquire the decreased expression of NR2F2 in BM-MSCs. The knock-down group was marked knock-down and the scrambled control group was marked Neg hereafter. The transfected cells expressed the GFP protein as shown in Figures 2(b) and 2(d). As shown in Figure 2(e), the transfected BM-MSCs in both groups were positive for CD90, CD105, and CD73 but were negative for CD45, CD34, and CD19. The decreased expression of NR2F2 was confirmed by western blot analysis (Figure 2(f)).

### 3.2. The Knock-Down of NR2F2 Suppressed the Colony-Forming Capacity of BM-MSCs

**Figure 3:**
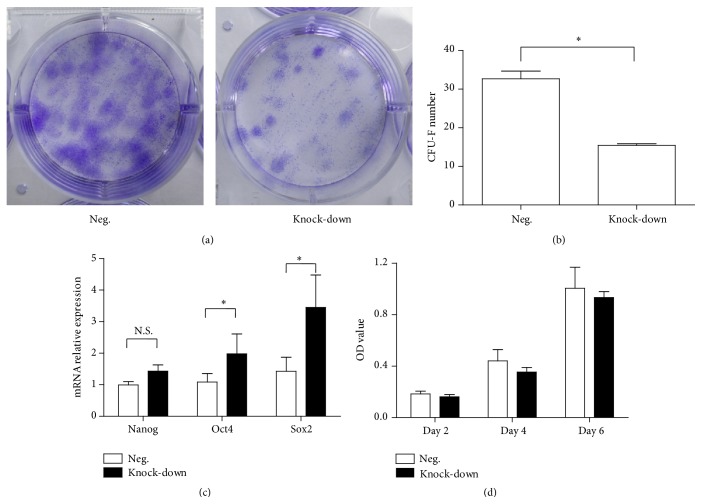
Self-renewal of transfected BM-MSCs. (a) Representative CFU-F of transfected BM-MSCs in a 6-well plate. (b) Mean value (±SD) of CFU-F number in different groups. (c) Mean relative values (±SD) of* NANOG*,* OCT4*, and* SOX2* mRNA expression in different groups. (d) Mean value (±SD) of OD values in different groups detected by MTT assay.

The renewal efficiency of BM-MSCs was evaluated by the rate of colony formation in colony-forming unit-fibroblast (CFU-F) assay. The number of CFU-F of BM-MSCs in knock-down group was significantly less than that in negative control (15.45 ± 0.39 versus 32.67 ± 2.03, *P* < 0.05, Figures 3(a) and 3(b)). We also evaluated the expression of pluripotency genes including* NANOG*,* OCT4*, and* SOX2* that were expressed in MSCs and were indispensable for maintaining pluripotency and self-renewal. Inconsistent with the results of CFU-F assay, the relative mRNA expression of* NANOG*,* OCT4*, and* SOX2* increased in the knock-down group compared with the negative control, especially for* OCT4* and* SOX2*, which achieved significant differences. In addition, we utilized MTT assay to evaluate the proliferation of BM-MSCs at 2 days, 4 days, and 6 days (Figure 3(c)). Although there were no significant differences, the OD values were lower in the knock-down group. These results revealed that decreased expression of NR2F2 suppressed the capacity of colony formation but did not reduce the expression of pluripotency genes (Figure 3(d)).

### 3.3. The Knock-Down of NR2F2 Suppressed the Osteogenic and Adipogenic Differentiation of BM-MSCs

**Figure 4:**
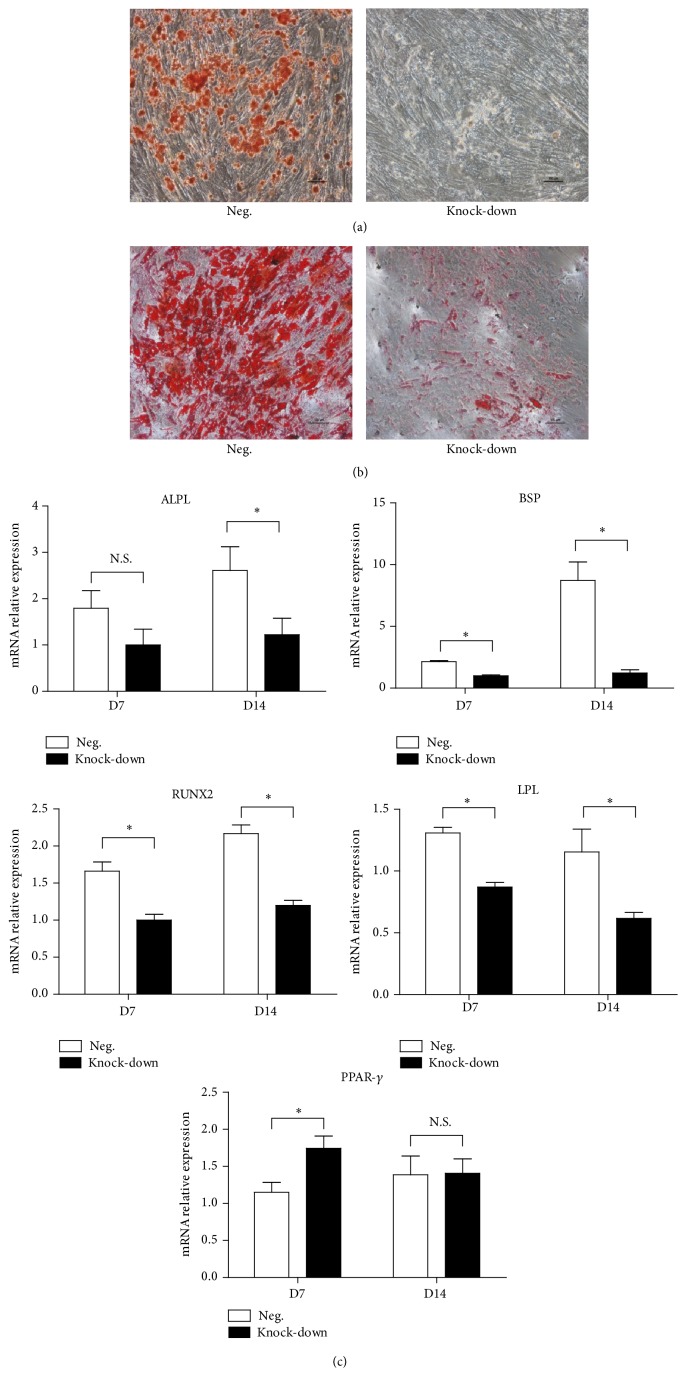
Osteogenic and adipogenic differentiation of transfected BM-MSCs. (a) Osteogenic differentiation of transfected BM-MSCs was detected by Alizarin Red staining. Scale bar = 100 *μ*m. (b) Adipogenic differentiation of transfected BM-MSCs was demonstrated via Oil red O staining. Scale bar = 100 *μ*m. (c) Mean relative values (±SD) of* ALPL*,* BSP*,* RUNX2*,* LPL*, and* PPAR-γ* mRNA expression in Day 7 and Day 14 of induction culture processes.

To evaluate whether the knock-down of NR2F2 had effects on osteogenesis of BM-MSCs, both groups of transfected BM-MSCs were cultured with osteogenic induction medium for 21 days. The staining of Alizarin Red showed that MSCs in knock-down group exhibited less calcium deposition than those in negative control. The mRNA expression levels of* BSP* and* RUNX2* markedly decreased in the knock-down group compared with control at day 7 and day 14 (Figure 4(a)) and the mRNA expression level of* ALPL* decreased at day 14 (Figure 4(c)).

As for adipogenic differentiation of BM-MSCs, both groups of cells were cultured in adipogenic induction medium according to manufacturer's instruction. MSCs in the knock-down group showed reduced Oil red O+ staining compared with the control group. The mRNA expression level of* LPL* markedly decreased in the knock-down group compared with the control group at day 7 and day 14 (Figure 4(b)). However, the mRNA expression level of* PPAR-γ* did not decrease at day 7 or day 14. Through these experiments, we confirmed that knock-down of NR2F2 suppressed the osteogenic and adipogenic differentiation of BM-MSCs (Figure 4(c)).

### 3.4. The Knock-Down of NR2F2 Accelerated Senescence of BM-MSCs but Had No Effects on Apoptosis of BM-MSCs

**Figure 5:**
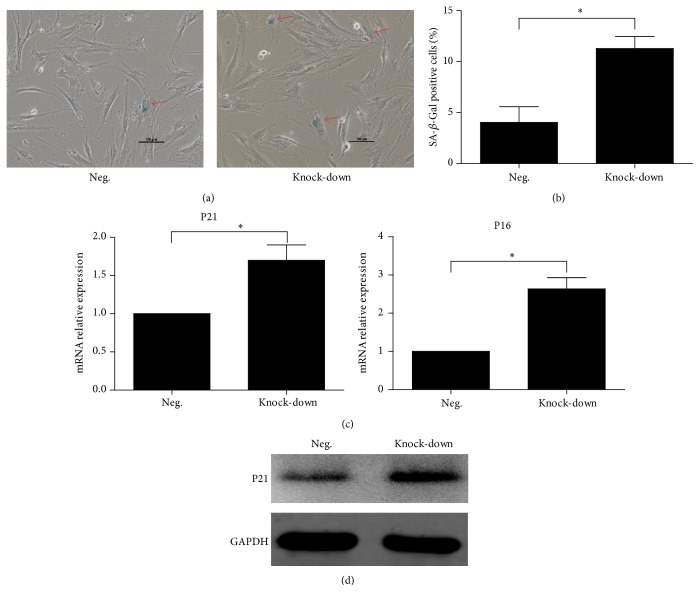
Senescence analysis of transfected BM-MSCs. (a) Representative SA-*β*-Gal activity in different groups. Scale bar = 100 *μ*m. (b) Percentage of SA-*β*-Gal positive cells was quantified in different groups. Data was presented as mean ± SD. (c) Mean relative values (±SD) of* P21* and* P16* mRNA expression in different groups. (d) The confirmation of P21 upregulation in knock-down group by western blot analysis.

**Figure 6:**
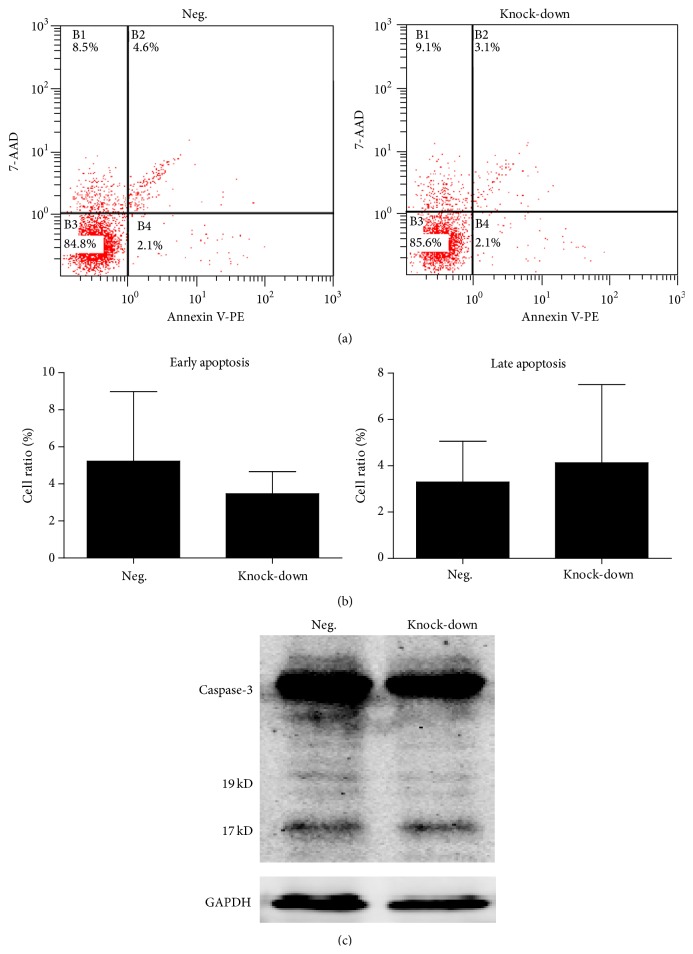
Apoptosis analysis of transfected BM-MSCs. (a) Representative flow cytometric results of apoptosis analysis in different groups. (b) Percentage of early and late apoptosis cells in different groups. Data was presented as mean ± SD. (c) Western blot analysis of Caspase-3 in different groups. The 17 kD and 19 kD bands represented cleaved Caspase-3.

Subsequently, we evaluated the senescence of transfected BM-MSCs. The staining of senescence-associated *β*-galactosidase (SA-*β*-Gal) revealed that the knock-down group displayed a higher percentage of senescent cells. We also detected that the mRNA expression of* P21* and* P16* increased (Figures 5(a) and 5(b)), which was consistent with the SA-*β*-Gal staining results. The elevated expression of P21 was also confirmed by western blot analysis (Figure 5(c)). We also assessed the apoptosis of transfected BM-MSCs (Figure 5(d)). The results of flow cytometry assays revealed no significant differences between the knock-down group and the negative control group. The western blot analysis of Caspase-3 showed similar results in both groups (Figures 6(a) and 6(b)).

## 4. Discussion

In this study, we explored the role of NR2F2 in the regulation of biological characteristics of BM-MSCs via the shRNA-mediated knock-down of NR2F2. Our results revealed that the suppression of NR2F2 via knock-down decreased the CFU-F efficiency of BM-MSCs. The generation of CFU-F in culture reflected the self-renewal capacity, which referred to the proliferation of stem cells without lineage differentiation [12]. Therefore, our results indicated that the suppression of NR2F2 affected self-renewal capacity of BM-MSCs. However, the expression of pluripotency genes exhibited inconsistent results.* NANOG*,* SOX2*, and* OCT4* were proposed as transcription factors that regulated the maintenance of the pluripotent state in embryonic stem cells and adult stem cells [13, 14]. Later research proposed distinct results that NANOG, but not OCT4 or SOX2, regulated pluripotency in human MSCs [15]. In our results, after suppression of NR2F2, the mRNA expression of* NANOG*,* OCT4*, and* SOX2* all increased, although the increase of* NANOG* had no significance. It has been reported that the suppression of NR2F2 played a role in the upregulation of OCT4, which was partly consistent with our results [16]. Our data suggested that effects of NR2F2 on self-renewal capacity were not regulated by these pluripotency genes. In addition, the results of CFU-F assay reflected that the suppression of NR2F2 inhibited the long-term proliferation of BM-MSCs in 14 days. Therefore, we evaluated the short-term proliferation of BM-MSCs in both groups in 2 days, 4 days, and 6 days. After indicated days of incubation, although there were no significant differences, the OD values of the knock-down group were all lower than those of the negative control group. These results indicated that the suppression of NR2F2 may induce a decreasing trend of short-term proliferation of BM-MSCs.

Consequently, we studied the effects of NR2F2 suppression on senescence and apoptosis of BM-MSCs. The senescence analysis revealed that knock-down of NR2F2 led to a higher proportion of SA-*β*-Gal positive cells. The apoptosis analysis suggested no significant differences between the two groups. These results indicated that the decrease of CFU-F was partly attributed to the acceleration of senescence. Subsequently, we evaluated the expression of* P21* and* P16*, which were proposed to initiate senescence via inhibition of CDK4/6 [17]. Our data revealed that both were elevated. The increase of P21 after NR2F2 suppression was consistent with previous report, which proposed that overexpression of NR2F2 downregulated P21 through direct association with SMAD4 [18]. Whether the regulation of P21 by NR2F2 was attributed to the direct association with SMAD4 in our case requires further research. Taken together, the self-renewal capacity and proliferation affected by NR2F2 suppression may be attributed to the acceleration of senescence through the upregulation of P21 and P16.

We also evaluated the effects of NR2F2 suppression on adipogenesis and osteogenesis of BM-MSCs. Both differentiation processes were notably suppressed according to the staining results after corresponding induction culture. Previous reports revealed that loss of NR2F2 induced impaired adipogenic program and intensive osteogenesis [10]. Although the switching of cell fate decision from adipocytes to osteoblasts by suppressing the transactivation function of PPAR-gamma has been demonstrated [19], another report suggested conflicting results [20]. The expression of* PPAR-γ* did not decrease in our case; however the decrease of* LPL* and reduced Oil red O+ staining still referred to suppression of adipogenesis. Since we utilized the same sequence of shRNA as previously reported [10, 11], reports of inconsistent results may be attributed to different species of MSCs. Previous conclusions [10, 21] were achieved by experiments done in mice, mice-derived MSCs, or mice cell lines. Our data was based on human bone marrow-derived MSCs. On the other hand, our results showed the inhibition of proliferation in BM-MSCs, which may also affect the osteogenic differentiation. Future studies of detailed mechanisms of different results are needed.

## 5. Conclusion

In conclusion, our results have demonstrated that the suppression of NR2F2 by shRNA-mediated knock-down impaired self-renewal capacity and pluripotency of bone marrow-derived mesenchymal stem cells. The inhibition of colony-forming capacity may be attributed to the acceleration of senescence through upregulation of P21 and P16. Therefore, NR2F2 plays an important role in the stemness maintenance of BM-MSCs.

## Abbreviations

MSCs: Mesenchymal stem cells
BM-MSCs: Bone marrow-derived mesenchymal stem cells
CFU-F: Colony-forming unit-fibroblasts
NR2F2: Nuclear receptor subfamily 2, group F, member 2
GVHD: Graft-versus-host disease
SA-*β*-Gal: Senescence-associated *β*-galactosidase
GAPDH: Glyceraldehydes 3-phosphate dehydrogenase
ALPL: Alkaline phosphatase
BSP: Binding sialoprotein
RUNX2: Runt-related transcription factor 2
LPL: Lipoprotein lipase
PPAR-*γ*: Peroxisome proliferator-activated receptor-*γ*.
